# Large‐scale pathogenicity prediction analysis of cancer‐associated kinase mutations reveals variability in sensitivity and specificity of computational methods

**DOI:** 10.1002/cam4.6324

**Published:** 2023-07-06

**Authors:** Sravani Akula, Sai Charitha Mullaguri, Niklas Max Melton, Archana Katta, Venkata Sai Giridhar Reddy Naga, Shyamson Kandula, Raj Kumar Pedada, Janakiraman Subramanian, Rama Krishna Kancha

**Affiliations:** ^1^ Molecular Medicine and Therapeutics Laboratory, CPMB Osmania University Hyderabad India; ^2^ Thoracic Oncology, Inova Schar Cancer Institute Fairfax Virginia USA; ^3^ Applied Computational Intelligence Lab Missouri University of Science and Technology Rolla Missouri USA

**Keywords:** kinase, pathogenicity prediction, point mutations, prediction accuracy, sensitivity and specificity

## Abstract

**Background:**

Mutations in kinases are the most frequent genetic alterations in cancer; however, experimental evidence establishing their cancerous nature is available only for a small fraction of these mutants.

**Aims:**

Predicition analysis of kinome mutations is the primary aim of this study. Further objective is to compare the performance of various softwares in pathogenicity prediction of kinase mutations.

**Materials and methods:**

We employed a set of computational tools to predict the pathogenicity of over forty‐two thousand mutations and deposited the kinase‐wise data in Mendeley database (Estimated Pathogenicity of Kinase Mutants [EPKiMu]).

**Results:**

Mutations are more likely to be drivers when being present in the kinase domain (vs. non‐kinase domain) and belonging to hotspot residues (vs. non‐hotspot residues). We identified that, while predictive tools have low specificity in general, PolyPhen‐2 had the best accuracy. Further efforts to combine all four tools by consensus, voting, or other simple methods did not significantly improve accuracy.

**Discussion:**

The study provides a large dataset of kinase mutations along with their predicted pathogenicity that can be used as a training set for future studies. Furthermore, a comparative sensitivity and selectivity of commonly used computational tools is presented.

**Conclusion:**

Primary‐structure‐based in silico tools identified more cancerous/deleterious mutations in the kinase domains and at the hot spot residues while having higher sensitivity than specificity in detecting deleterious mutations.

## 1 INTRODUCTION

Mutated kinases are highly sought‐after therapeutic targets. Next‐generation sequencing (NGS) methods have identified a variety of somatic mutations in kinases across multiple cancers; however, the functional significance and pathogenic nature of less‐frequent mutations is largely unknown.^1^ It is important to understand the role of mutations in tumorigenesis in order to determine the treatment strategy using targeted therapeutics.^2^ Therefore, classification of many rare kinase mutations as either driver or passenger mutations is an important step for precision oncology. It is difficult, however, to experimentally determine the cancerous nature of each of these rare mutations^3^ thus stressing the need for the prediction of their pathogenicity. Importantly, experimental validation of about 3400 mutations that were predicted as pathogenic in a pan‐cancer and pan‐software study concluded that 60–85% are likely drivers highlighting the utility of pathogenicity prediction analyses for less frequent mutations.^4^

While several studies have reported computational methods for predicting the pathogenic nature of rare mutations, studies on predicting the pathogenicity of kinase mutants are scant. A previous study using SIFT and PolyPhen‐2 tools reported the enrichment of somatic mutations in phosphorylation sites as effecting kinase‐substrate interactions and indicating deleteriousness.^5^ However, it remains important to predict the extent of pathogenicity of all kinase mutations irrespective of their ability to be phosphorylated. Another recent study by Rodrigues et al. reported a computational approach in which the pathogenicity of kinase mutations was predicted with high accuracy although only for a small set of mutations.^6^ It was also reported that tumorigenic activating mutations tended to occur in kinase domain with a slightly higher selection pressure than those in non‐kinase domains.^7^ With the routine use of NGS testing of tumor tissue in both the clinic and the laboratory, identifying the functional significance for many of these mutations is a major hurdle. In light of this, we collected kinase mutants reported in the Catalog of Somatic Mutations in Cancer (COSMIC) database, predicted their pathogenic nature using multiple in silico tools, performed comparative analysis across the kinome, and identified factors that determine the likelihood of a mutation to be a driver. Further, we have estimated the accuracy of the individual primary structure tools to help clinicians and research scientists interpret their own results when using these tools.

## 2 MATERIALS AND METHODS

Four widely used in silico tools were selected to predict the pathogenicity of mutations in the kinome. PolyPhen‐2 (Polymorphism Phenotyping v2, a Naïve Bayes Classifier based method) combines sequence‐based and structure‐based features.^8^ SIFT (Sorting Intolerant From Tolerant) is a position specific scoring matrix‐based method which predicts the deleterious nature of mutations according to the sequence homology from the PSI‐BLAST method.^9^ PredictSNP is a consensus classifier based on eight prediction tools including PolyPhen‐2 and SIFT.^10^ FATHMM (Functional Analysis Through Hidden Markov Model) predicts cancerous nature of mutations based on the sequence homology.^11^ Our cancer kinome dataset included 42,165 point mutations belonging to 248 kinases from the COSMIC database. In total, 248 excel files were deposited in Mendeley database (10.17632/xn3xrppsyy.1), each containing prediction data for kinase mutations based on SIFT, PolyPhen‐2, PredictSNP, and FATHMM.

To estimate performance of each tool, we evaluated accuracy, sensitivity, and specificity^12^ on a ground‐truth dataset composed of 141 kinase mutations (99 cancerous and 42 inert) with experimentally proven activity. As there are very few examples of inert kinase mutations in the literature, there was no alternative to the ground‐truth dataset with a class imbalance. For this reason, we emphasize the balanced accuracy metric in our evaluation.

Accuracy:

$$\frac{\text{True Positive}\;\left[\mathrm{TP}\right]+\text{True Negative}\;\left[\mathrm{TN}\right]}{\text{True Positive}\;\left[\mathrm{TP}\right]+\text{True Negative}\;\left[\mathrm{TN}\right]+\text{False Positive}\;\left[\mathrm{FP}\right]+\text{False Negative}\;\left[\mathrm{FN}\right]}.$$

Sensitivity:

$$\frac{\mathrm{TP}}{\mathrm{TP}+\mathrm{FN}}.$$

Specificity:

$$\frac{\mathrm{TN}}{\mathrm{TN}+\mathrm{FP}}.$$

Balanced Accuracy:

$$\frac{\text{Sensitivity}+\text{Specificity}}{2}.$$

We further analyzed whether the balanced accuracy of the individual in silico tools could be improved on by leveraging either a consensus or voting method derived from all four tools. Additionally, a total of 12,544 stability predictions for mutations spanning 100 kinases were gathered using the tertiary structure‐based tools CUPSAT,^13^ SDM,^14^ mCSM,^15^ and DynaMut.^16^ We tested the accuracy, sensitivity, and specificity of these tertiary tools against 119 kinase mutations (99 cancerous and 20 inert) with known activity. Influenced by the variability in protein structure reported in protein databases (PDBs), we used protein structure from two different PDBs for each of the 119 kinase mutations. Concordance (count of pairs with same result/total counts of paired results) for the paired results reported by each tool were then calculated to understand the response for each tool. Kinome trees depicting our analyses were built using KinHub software as described previously.^17^

## 3 RESULTS AND DISCUSSION

**FIGURE 1:**
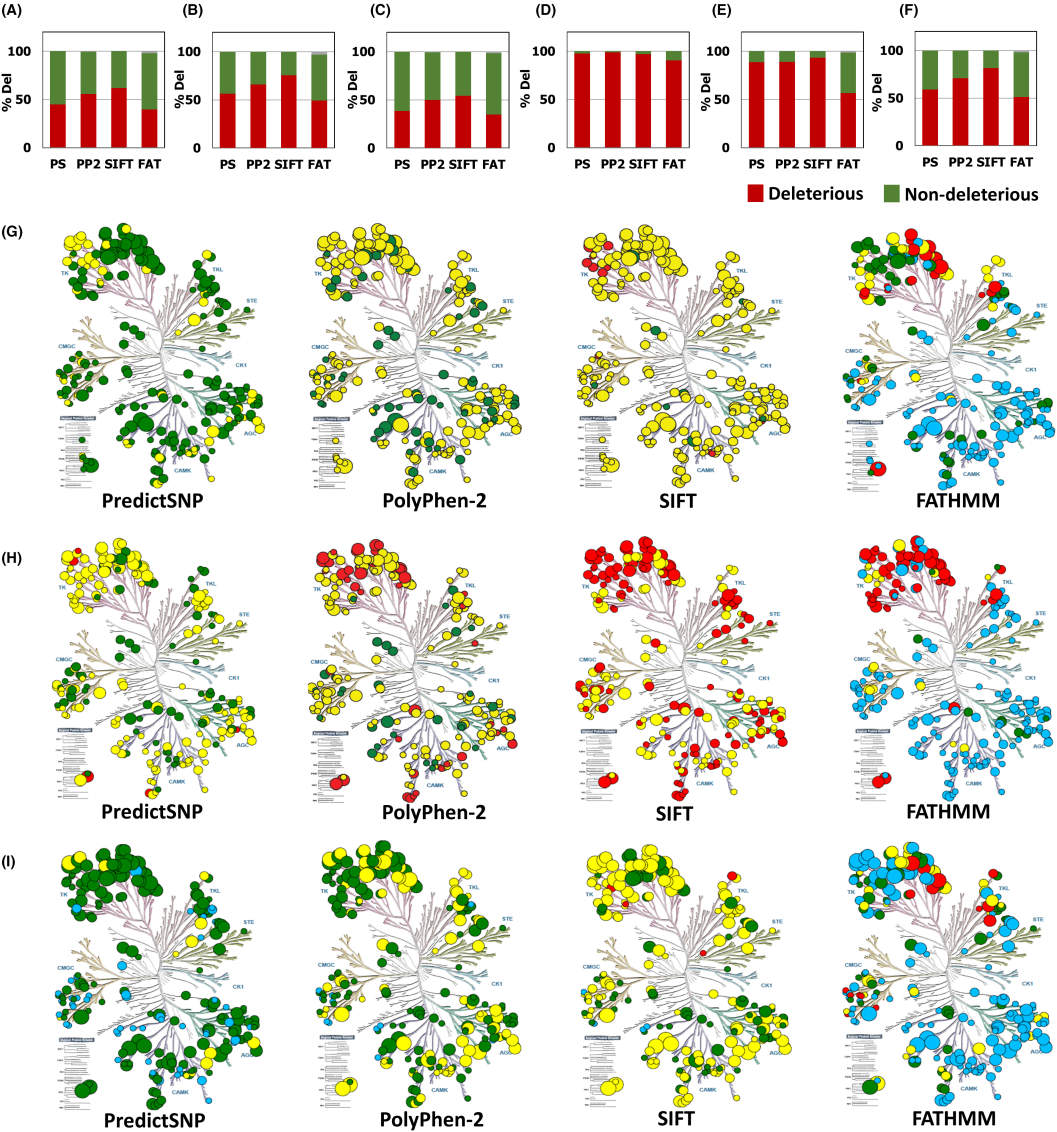
Predicted pathogenicity of kinase mutants by four primary structure‐based tools. (A) Pathogenicity predictions of kinase mutants using primary structure‐based tools. (B–F) Region/sub‐region‐specific analysis: kinase domain (B), non‐kinase domain (C), DFG‐motif (D), p‐loop (E) and alpha‐C‐helix (F). Percentage of mutations that were predicted to be deleterious/cancerous in all kinases were shown in red while percentage of predicted neutral mutations were shown in green. Distribution of total number of mutations (bubble size) as well as the percentage of deleterious/cancerous mutations (bubble color) for complete kinase (G), kinase domain (H), and the non‐kinase domain (I) are shown. Bubble size represents the number of mutations studied for that particular kinase. Large: >250 mutations/kinase; intermediate: 101–250 mutations/kinase; small: 50–100 mutations/kinase and very small: <50 mutations/kinase. Bubble color represents the percentage of deleterious mutations for that particular kinase. Red: 75.1–100% deleterious mutations; yellow: 50.1–75% deleterious mutations; green: 25.1–50% deleterious mutations; blue: ≤25% deleterious mutations. PS: PredictSNP, PP2: PolyPhen‐2 and FAT: FATHMM.

Of the four primary structure tools, FATHMM could predict the cancerous nature of 38,483 mutations out of 42,165 total mutations. For the rest, wild type inconsistency was the reason for the lack of prediction. The proportion of deleterious mutations predicted was highest for SIFT at 61.8% while PolyPhen‐2 closely followed at 55.6%. On the contrary, FATHMM predicted the least at 39.4% while PredictSNP predicted marginally more at 44.9% (Figure 1A). Using a dataset of 1036 cancer‐associated somatic mutations in kinases, Torkamani et al. reported that nearly half (49.42%) of the mutations as drivers.^18^ Similarly, the in silico tools used in the present study also predicted approximately 40–60% of mutations to be pathogenic albeit on a much larger dataset further corroborating that less‐frequent mutations can be drivers too.^19^ Previously, annotation of mutations as drivers or passengers (neutral) based on a domain mutational landscape approach proved superior over the traditional gene landscape approach in colorectal and breast cancers.^20^ Here, we find that a higher percentage of mutations in the kinase domain (KD) (Figure 1B) rather than the non‐kinase domain (NKD) (Figure 1C) were predicted to be deleterious unanimously by all four primary structure tools (Figure S1). This observation also confirms a previous report wherein 66.67% of driver mutations belonged to the catalytic domain.^18^ Further analysis of sub‐regions within the KD revealed a higher percentage of deleterious/cancerous mutations in DFG motif (Figure 1D) followed by in p‐loop (Figure 1E) and in a‐C helix (Figure 1F). Notably, mutations occurring in p‐loop were previously shown to exhibit high selection pressure.^7^ These results thus indicate a correlation between pathogenicity and functional modularity of kinases.

The proportion of predicted deleterious/cancerous mutations as compared to the total mutations per kinase varied among the kinases (Figure 1G). Among 248 kinases, more than half of the mutations were predicted to be deleterious in 60 (24.1%), 181 (72.9%), and 235 (94.7%) individual kinases by PredictSNP, PolyPhen‐2, and SIFT, respectively (Figure 1G). FATHMM predicted more than half of the mutations as cancerous in 53 out of 223 (23.7%) individual kinases (Figure 1G). Domain‐specific analysis revealed a significantly higher proportion of the KD mutations to be deleterious/cancerous (Figure 1H) than the NKD mutations (Figure 1I). If mutations at a residue involved more than one amino acid substitution, we considered the residue a hotspot (HS) and the mutations at that residue as HS mutations. A total of 127 kinases that harbored HS mutations were further analyzed, accounting for 6.6% (1957 mutations) of total number (29,415) of mutations within these kinases. The percentages of predicted deleterious/cancerous mutations were higher for HS than for non‐hotspot (NHS) mutations (Figure S2). The distribution of hotspot mutations (HS) among 127 kinases varied both in number and the percentage of predicted pathogenicity (Figure S2). Domain‐specific analysis revealed a higher percentage of HS mutations in the kinase domain (14.4%) than the non‐kinase domain (5.5%). The percentages of deleterious mutations were predicted to be higher among HS when compared with NHS mutations both in KD and NKD (Figure S3). The distribution of HS mutations among KD and NKD varied both in number and the percentage of predicted pathogenicity (Figure S3).

**FIGURE 2:**
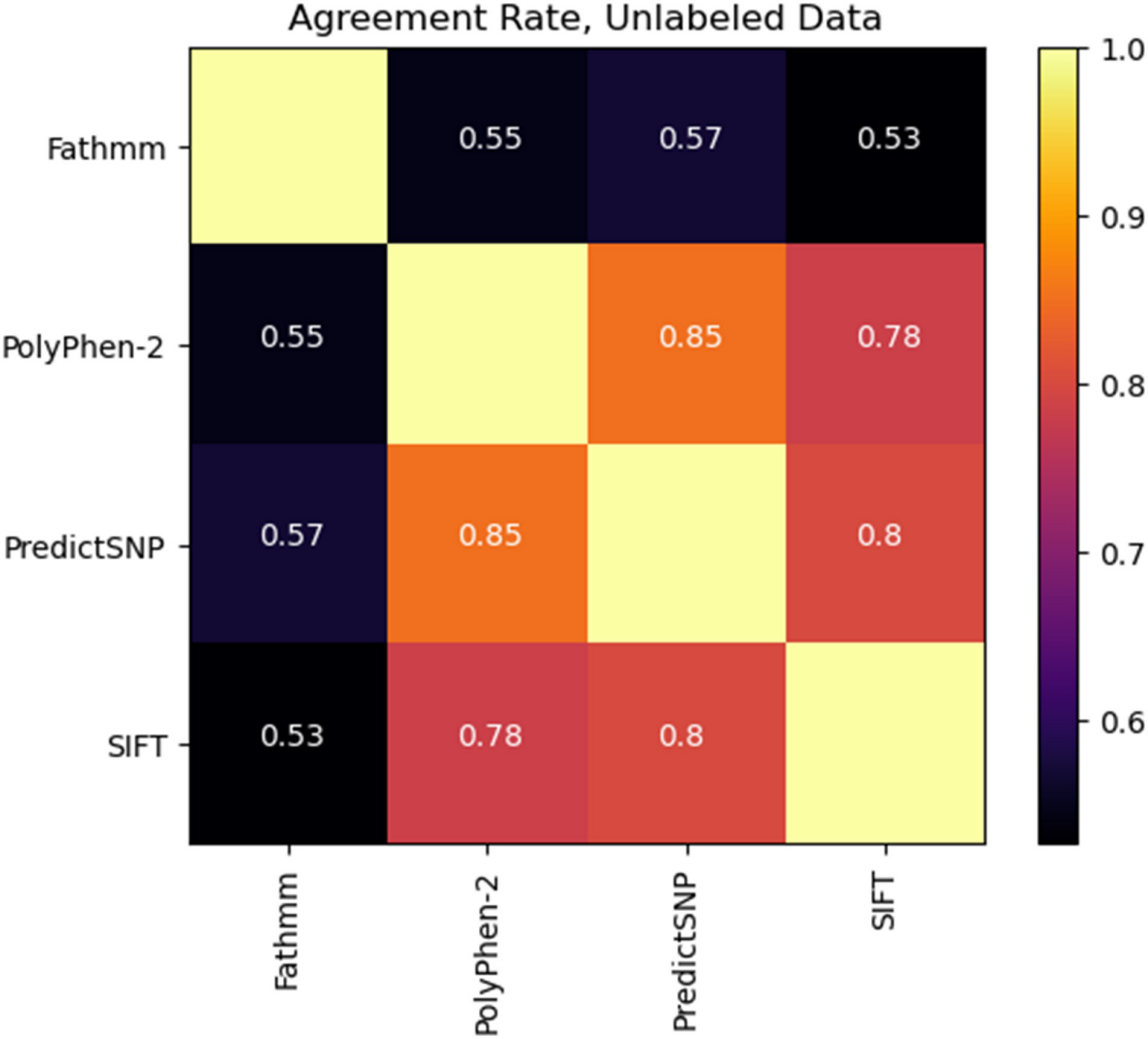
Agreement rates between individual primary tools. Heatmap showing the agreement of predictions made on the 42,165‐point mutations belonging to 248 kinases by individual primary structure‐based tools.

Analysis of agreements between all the four primary‐structure based primary tools revealed that one‐fifth of the mutations were predicted unanimously to be deleterious/cancerous (7435 mutations) or neutral/passenger (8894 mutations). Importantly, agreement rate was higher among PolyPhen‐2, SIFT and PredictSNP but not with FATHMM (Figure 2 and Figure S4). A higher percentage of mutations with 100% consensus were seen for tyrosine kinases belonging to RTK, NRTK, and TKL families (Figure S5). However, a significant variability was observed between individual members within each kinase family (Figure S5). Among individual kinases, the highest percentage of mutations with 100% consensus was observed in GSK3A (48.5%), EGFR (47.1%), and JNK1 (46%) (Figure S5).

Further, to assess the accuracy of individual primary tools, we analyzed a panel of 99 oncogenic mutants belonging to eight kinases which were previously shown to transform Ba/F3 cells to cytokine independence as well as 42 inactive mutants/variants (Figure S6). The measured sensitivity was 90.4%, for FATHMM, 86.9% for PolyPhen‐2, 75.7% for SIFT, and 63.6% for PredictSNP (Table S1). FATHMM exhibited by far the lowest specificity at 11.9% compared to 45.2%, 52.4%, and 59.5% for PolyPhen‐2, SIFT, and PredictSNP, respectively (Table S1). Based on the COSMIC dataset, PolyPhen‐2 (79%) was previously shown to have higher sensitivity than the SIFT (70%), but, SIFT (82%) showed higher specificity than that of the PolyPhen‐2 (75%).^21^ However, based on the COBR dataset, SIFT (73%) showed higher sensitivity than PolyPhen‐2 (63%) and PolyPhen‐2 (66%) showed higher specificity than the SIFT (55%). Therefore, the sensitivity and specificity of prediction softwares varied depending on the dataset and the gene under consideration. In another example, PolyPhen‐2 displayed higher sensitivity and FATHMM showed higher specificity both with VariBench and SwissVar benchmarking datasets.^11^ Interestingly, SIFT showed maximum sensitivity for mutations in *BRCA1* and *MSH2* genes, PolyPhen‐2 for *MSH2* and *TP53* genes, and FATHMM for *MLH1* and *TP53* genes.^11^ On the contrary, PolyPhen‐2 displayed maximum specificity for mutations in *MLH1* and *TP53* genes while FATHMM for *BRCA1* and *MSH2* genes.^11^ Given the imbalance in sample sizes between the active and inactive kinases, we calculated balanced accuracy as being 66.1%, 64.1%, 61.6%, and 51.1% for PolyPhen‐2, SIFT, PredictSNP, and FATHMM respectively. We further conducted consensus and voting methods of all four tools (Table S2) in hope of achieving higher prediction accuracy but neither method significantly improved balanced accuracy over PolyPhen‐2. Considering the limited number of experimentally confirmed kinase mutation data and the implied complexity of performing a consensus or voting approach, PolyPhen‐2 seems to be the best choice followed by PredictSNP and SIFT. FATHMM had the lowest balanced accuracy of all the four tools. Overall, our observations with the kinome dataset are in line with a previous study that ranked PolyPhen‐2 among the best prediction tools followed by the SIFT among the medium and FATHMM among the low performing tools.^22^

We additionally used four tertiary structure‐based tools to predict the stabilizing/destabilizing effects of kinase mutants. A total of 12,544 predictions for about 6626 mutations that spanned kinase domains of 100 kinases for which (co)‐crystal structures are available and were considered for this analysis. The percentage of mutations that were predicted as destabilizing varied widely between the four tertiary tools: CUPSAT (67.1%), mCSM (78.2%), SDM (80.9%), and DynaMut (32.2%). We have identified several limitations with the utility of tertiary structure‐based tools: (1) several mutations did not map to a solved crystal structure; (2) structural differences between multiple PDBs could result in discordant pathogenicity predictions, and currently, there is no directive principle to choose a particular PDB for a specific protein; (3) several instances were identified where different PDBs for the same gene mutation gave discordant results; and (4) a prediction of “destabilizing” by tertiary tools may not necessarily mean deleterious/cancerous. In the test for concordance, mCSM has the highest concordance rate (91.6%) between the two PDBs for a given protein followed by SDM (76.5%), CUPSAT (71.4%), and DynaMut (57.1%). For subsequent assessment of accuracy, we tested mCSM alone given the lower concordance for the other three tertiary tools. The sensitivity for mCSM was high at 85.8% while specificity was low at 20%. Using a dataset of 384 mutations within 42 kinases, the web server Kinact was shown to perform better than mCSM.^6^ However, Kinact again relies on the tertiary structure of the protein and many kinases still lack a solved structure.^6^ A comparative study of 989 mutations concluded that the accuracy of prediction softwares varied considerably and suggested that combinations of methods might improve the prediction performance.^23^ Therefore, we combined the best performing primary structure based PolyPhen‐2 with the mCSM and observed that the sensitivity improved to 97.9% and specificity to 54.8%.

Taken together, our study indicates that the likelihood of a kinase mutant to be cancerous increases if its location is both in the kinase domain (vs non‐kinase domain) and is a hot‐spot mutation (vs non‐hot spot mutation). Among all in silico tools, PolyPhen‐2 provides the best balance in terms of accuracy, sensitivity, and specificity in identifying deleterious/cancerous mutations. Combining mCSM with PolyPhen‐2 does show promising results in our small testing cohort but further studies are needed to confirm the validity of this combination. The large compendium of kinase mutations with predicted pathogenicity collected by this study may be used as training datasets to validate future in silico prediction tools. Additionally, we hope the above conclusions may be of help to clinicians who identify rare kinase mutants in cancer patients.

## AUTHOR CONTRIBUTIONS

**Sravani Akula:** Data curation (equal); formal analysis (equal); investigation (equal); methodology (equal). **Sai Charitha Mullaguri:** Data curation (equal); formal analysis (equal); investigation (equal); methodology (equal). **Niklas Max Melton:** Data curation (equal); formal analysis (equal); investigation (equal); methodology (equal); resources (equal); validation (equal); writing – original draft (equal). **Archana Katta:** Data curation (equal); investigation (equal); methodology (equal). **Venkata Sai Giridhar Reddy Naga:** Data curation (equal); investigation (equal); methodology (equal). **Shyamson Kandula:** Data curation (equal); investigation (equal); methodology (equal). **Raj Kumar Pedada:** Data curation (equal); investigation (equal); methodology (equal). **Janakiraman Subramanian:** Conceptualization (equal); investigation (equal); project administration (equal); supervision (equal); validation (equal); writing – original draft (equal); writing – review and editing (equal). **Rama Krishna Kancha:** Conceptualization (equal); data curation (equal); formal analysis (equal); funding acquisition (lead); investigation (equal); project administration (lead); supervision (lead); validation (equal); writing – original draft (equal); writing – review and editing (equal).

## CONFLICT OF INTEREST STATEMENT

SA, SCM, NMM, AK, VSGRN, SK, RKP, and RKK have no relevant financial or non‐financial interests to disclose.

JS: Consulted for AstraZeneca, Boehringer Ingelheim, Pfizer, Novartis, Daichi, G1 Therapeutics, Jazz Pharmaceuticals, Janssen Oncology, Lilly, Blueprint medicines, Axcess, BeiGene, Cardinal Health, Takeda, and OncoCyte. Participated as a speaker for AstraZeneca, Boehringer Ingelheim, G1 therapeutics, Jazz Pharmaceuticals, and Janssen Oncology.

## 4 AUTHOR CONTRIBUTIONS

Sravani Akula: Data curation (equal); formal analysis (equal); investigation (equal); methodology (equal). Sai Charitha Mullaguri: Data curation (equal); formal analysis (equal); investigation (equal); methodology (equal). Niklas Max Melton: Data curation (equal); formal analysis (equal); investigation (equal); methodology (equal); resources (equal); validation (equal); writing—original draft (equal). Archana Katta: Data curation (equal); investigation (equal); methodology (equal). Venkata Sai Giridhar Reddy Naga: Data curation (equal); investigation (equal); methodology (equal). Shyamson Kandula: Data curation (equal); investigation (equal); methodology (equal). Raj Kumar Pedada: Data curation (equal); investigation (equal); methodology (equal). Janakiraman Subramanian: Conceptualization (equal); investigation (equal); project administration (equal); supervision (equal); validation (equal); writing—original draft (equal); writing—review and editing (equal). Rama Krishna Kancha: Conceptualization (equal); data curation (equal); formal analysis (equal); funding acquisition (lead); investigation (equal); project administration (lead); supervision (lead); validation (equal); writing—original draft (equal); writing—review and editing (equal).

## ETHICS STATEMENT

This is a data analysis study; therefore, no ethical approval is required.

## Supporting information


**Data S1:** Supporting Information.

## Data Availability

The data generated during and/or analysed during the current study are available from the corresponding author on request.
