# Supplementary material for: Large‐scale pathogenicity prediction analysis of cancer‐associated kinase mutations reveals variability in sensitivity and specificity of computational methods
Source: Cancer Med. 2023 Jul 6;12(16):17468–74. doi: 10.1002/cam4.6324 (PMC10501281; doi:10.1002/cam4.6324)
Supplement: Supplementary file 1 — Data S1: Supporting Information. [file CAM4-12-17468-s001.pdf]

Supplementary Table 1: The prediction accuracies, sensitivities and specificities of primary structure based and tertiary structure based tools on 99 oncogenic mutants and 42 inert mutants/variants

Method	Accuracy	Balanced Accuracy	True Positive Rate	False Positive Rate	True Negative Rate	False Negative Rate	Positve Predictive Value	Negative Predictive Value	Sensitivity	Specificity	Positive F1	Negative F1	Positive Samples	Negative Samples
Fathmm	0.66176	0.51165	0.90426	0.88095	0.11905	0.09574	0.696721	0.357143	0.9042553	0.119048	0.787	0.17857	94	42
PolyPhen-2	0.74468	0.66053	0.86869	0.54762	0.45238	0.13131	0.788991	0.59375	0.8686869	0.452381	0.8269	0.51351	99	42
PredictSNP	0.62411	0.6158	0.63636	0.40476	0.59524	0.36364	0.7875	0.409836	0.6363636	0.595238	0.7039	0.48544	99	42
SIFT	0.68794	0.64069	0.75758	0.47619	0.52381	0.24242	0.789474	0.478261	0.7575758	0.52381	0.7732	0.5	99	42
CUPSAT-1	0.63866	0.50354	0.70707	0.7	0.3	0.29293	0.833333	0.171429	0.7070707	0.3	0.765	0.21818	99	20
CUPSAT-2	0.65546	0.55354	0.70707	0.6	0.4	0.29293	0.853659	0.216216	0.7070707	0.4	0.7735	0.2807	99	20
SDM-1	0.58824	0.51313	0.62626	0.6	0.4	0.37374	0.837838	0.177778	0.6262626	0.4	0.7168	0.24615	99	20
SDM-2	0.63866	0.62323	0.64646	0.4	0.6	0.35354	0.888889	0.255319	0.6464646	0.6	0.7485	0.35821	99	20
DynaMut-1	0.36134	0.51641	0.28283	0.25	0.75	0.71717	0.848485	0.174419	0.2828283	0.75	0.4242	0.28302	99	20
DynaMut-2	0.52101	0.65227	0.45455	0.15	0.85	0.54545	0.9375	0.239437	0.4545455	0.85	0.6122	0.37363	99	20
mCSM-1	0.7479	0.52929	0.85859	0.8	0.2	0.14141	0.841584	0.222222	0.8585859	0.2	0.85	0.21053	99	20
mCSM-2	0.76471	0.53939	0.87879	0.8	0.2	0.12121	0.84466	0.25	0.8787879	0.2	0.8614	0.22222	99	20

Supplementary Table 2: The prediction accuracies, sensitivities and specificities of various algorithms on 99 oncogenic mutants and 42 inert mutants/variants

Method	Accuracy	Balanced Accuracy	True Positive Rate	False Positive Rate	True Negative Rate	False Negative Rate	Positve Predictive Value	Negative Predictive Value	Sensitivity	Specificity	Positive F1	Negative F1	Positive Samples	Negative Samples
Fathmm	0.66176	0.511651	0.90426	0.88095	0.11905	0.09574	0.6967213	0.357143	0.904255	0.119048	0.787037	0.178571	94	42
PolyPhen-2	0.74468	0.660534	0.86869	0.54762	0.45238	0.13131	0.7889908	0.59375	0.868687	0.452381	0.826923	0.513514	99	42
PredictSNP	0.62411	0.615801	0.63636	0.40476	0.59524	0.36364	0.7875	0.409836	0.636364	0.595238	0.703911	0.485437	99	42
SIFT	0.68794	0.640693	0.75758	0.47619	0.52381	0.24242	0.7894737	0.478261	0.757576	0.52381	0.773196	0.5	99	42
Consensus	0.62411	0.643218	0.59596	0.30952	0.69048	0.40404	0.8194444	0.42029	0.59596	0.690476	0.690058	0.522523	99	42
Vote	0.74468	0.65368	0.87879	0.57143	0.42857	0.12121	0.7837838	0.6	0.878788	0.428571	0.828571	0.5	99	42
Any	0.70213	0.520563	0.9697	0.92857	0.07143	0.0303	0.7111111	0.5	0.969697	0.071429	0.820513	0.125	99	42
Nearest Neighbors	0.60993	0.434343	0.86869	1	0	0.13131	0.671875	0	0.868687	0	0.757709		99	42
Linear SVM	0.70213	0.5	1	1	0	0	0.7021277		1	0	0.825		99	42
RBF SVM	0.70213	0.602814	0.84848	0.64286	0.35714	0.15152	0.7567568	0.5	0.848485	0.357143	0.8	0.416667	99	42
Gaussian Process	0.61702	0.487374	0.80808	0.83333	0.16667	0.19192	0.6956522	0.269231	0.808081	0.166667	0.747664	0.205882	99	42
Decision Tree	0.65957	0.606782	0.73737	0.52381	0.47619	0.26263	0.7684211	0.434783	0.737374	0.47619	0.752577	0.454545	99	42
Random Forest	0.64539	0.589827	0.72727	0.54762	0.45238	0.27273	0.7578947	0.413043	0.727273	0.452381	0.742268	0.431818	99	42
Neural Net	0.75177	0.645022	0.90909	0.61905	0.38095	0.09091	0.7758621	0.64	0.909091	0.380952	0.837209	0.477612	99	42
AdaBoost	0.66667	0.577561	0.79798	0.64286	0.35714	0.20202	0.745283	0.428571	0.79798	0.357143	0.770732	0.38961	99	42
Naive Bayes	0.58156	0.537518	0.64646	0.57143	0.42857	0.35354	0.7272727	0.339623	0.646465	0.428571	0.684492	0.378947	99	42
QDA	0.74468	0.667388	0.85859	0.52381	0.47619	0.14141	0.7943925	0.588235	0.858586	0.47619	0.825243	0.526316	99	42
Hist GB	0.65957	0.469697	0.93939	1	0	0.06061	0.6888889	0	0.939394	0	0.794872		99	42
Ridge	0.66667	0.577561	0.79798	0.64286	0.35714	0.20202	0.745283	0.428571	0.79798	0.357143	0.770732	0.38961	99	42
Ensemble	0.68794	0.613276	0.79798	0.57143	0.42857	0.20202	0.7669903	0.473684	0.79798	0.428571	0.782178	0.45	99	42

Supplementary figure 1: Schematic representation of regions within the kinase domain of EGFR kinase.

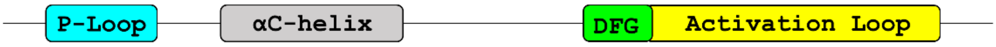


```
                                                P-Loop
MKKGHHHHHHDYDIPTTENLYFQGSGEAPNQALLRILKETEFKKIKVLGSGAFGTVYKGLWIPEGEKVKIPVA

       αC-helix
IKELREATSPKANKEILDEAYVMASVDNPHVCRLLGICLTSTVQLITQLMPFGCLLDYVREHKDNIGSQYLLN

                                                    Activation Loop
WCVQIAKGMNYLEDRRLVHRDLAARNVLVKTPQHVKITDFGLAKLLGAEEKEYHAEGGKVPIKWMALESILHR

IYTHQSDVWSYGVTVWELMTFGSKPYDGIPASEISSILEKGERLPQPPICTIDVYMIMVKCWMIDADSRPKFR

ELIIEFSKMARDPQRYLVIQGDERMHLPSPTDSNFYRALMDEEDMDDVVDADEYLIPQQG
```

Supplementary figure 2: Distribution of pathogenic hotspot mutations across the kinome. (A) Predicted pathogenicity of hotspot and non-hotspot mutations by primary structure-based tools. Percentage of predicted deleterious/cancerous and neutral mutations were represented in red and green, respectively. Percentage of hotspot (B) and non-hotspot (C) mutations for each kinase (bubble size) as well as the percentage of deleterious/cancerous mutations (bubble color) within the analyzed hotspot/non-hotspot mutations. Bubble size represents the percentage of hotspot and non-hotspot mutations in each kinase. Bubble size for hotspot mutations (HSMs), large: 25.1-50% HSMs/kinase; intermediate: 10.1-25% HSMs/kinase and small: 1-10% HSMs/kinase. Bubble size for non-hotspot mutations (NHSMs), large: 75.1-100% NHSMs/kinase; intermediate: 50.1-75% NHSMs/kinase; small: 25.1-50% NHSMs/kinase and very small: <u><</u>25% NHSMs/kinase. Bubble color represents the percentage of deleterious mutations within hotspot/non-hotspot mutations for that particular kinase. Red: 75.1-100% deleterious mutations; yellow: 50.1-75% deleterious mutations; green: 25.1-50% deleterious mutations; blue: <u><</u>25% deleterious mutations. PS – PredictSNP, PP2- PolyPhen-2, FAT – FATHMM.

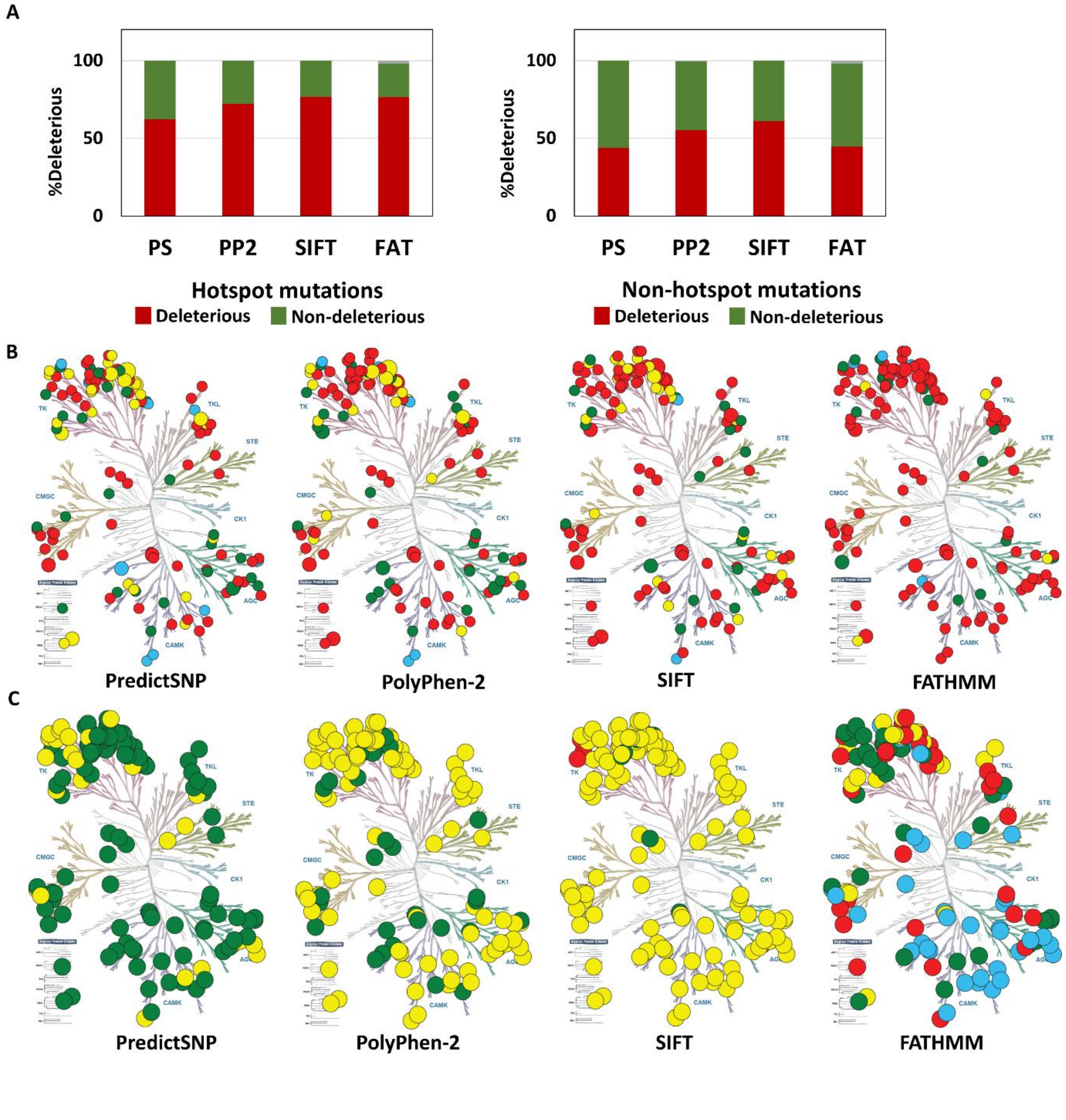

Supplementary figure 3: Domain-specific distribution of hotspot and non-hotspot mutations in the kinome. Percentage of hotspot mutations in the kinase domain (A) and non-kinase domain (C) as well as non-hotspot mutations in the kinase domain (B) and non-kinase domain (D) were represented as the bubble size. Large: 50.1-100% mutations/kinase; intermediate: 25.1-50% mutations/kinase; small: 10.1-25% mutations/kinase and very small: <u><</u>10% mutations/kinase. Bubble colour represents the percentage of deleterious/cancerous mutations within hotspot/non-hotspot mutations. Red: 75.1-100% deleterious mutations; yellow: 50.1-75% deleterious mutations; green: 25-50% deleterious mutations; blue: <25% deleterious mutations.

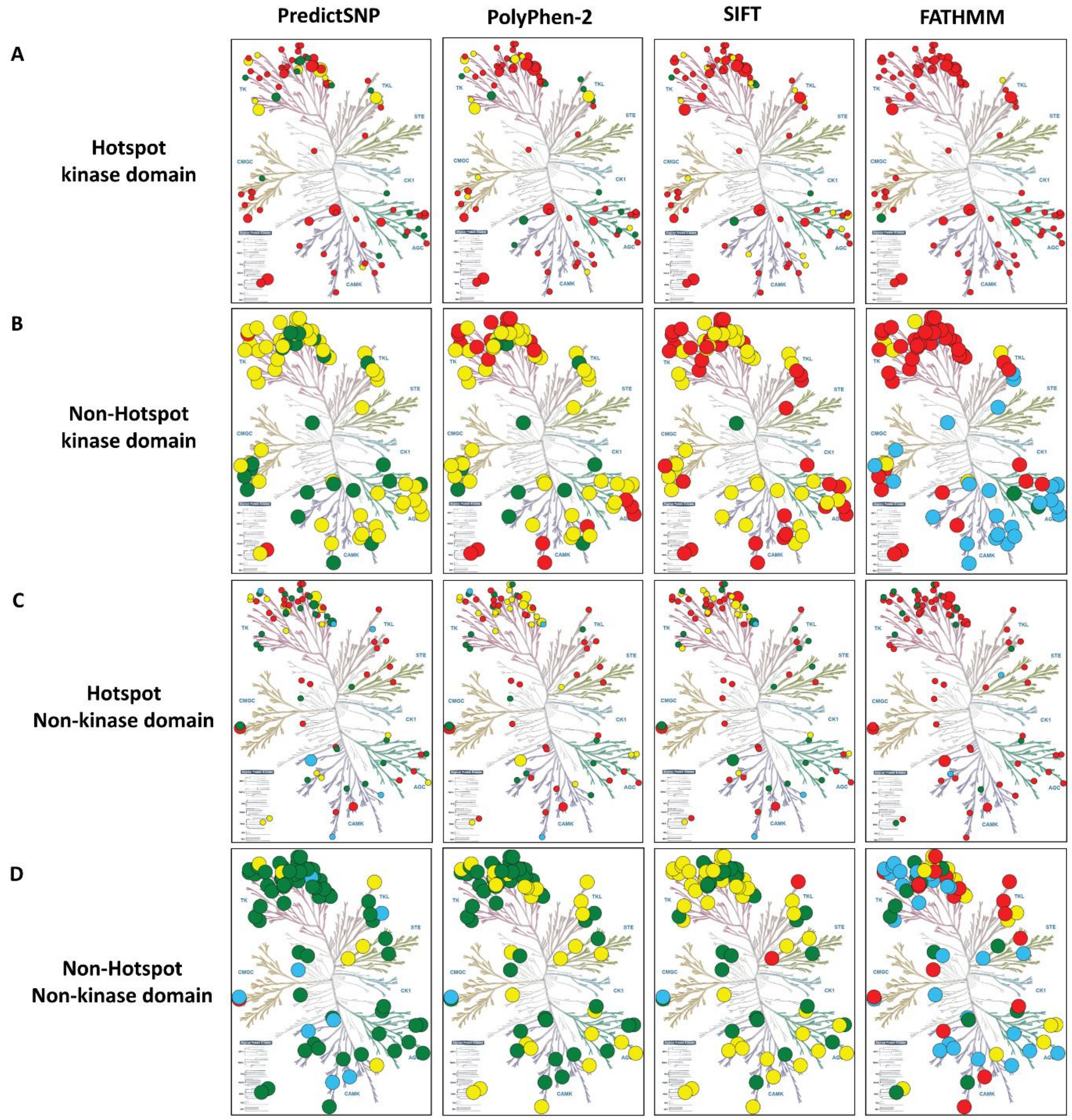

Supplementary figure 4: Percentage agreements for pathogenicity predictions between different primary structure based softwares. Correlation of predicted pathogenicity of kinase mutants between multiple primary structure based softwares. Domain/sub-domain-wise agreement between softwares is shown for kinase domain, non-kinase domain, P-loop, Alpha-C-helix and DFG-motif. PS – PredictSNP, PP2- PolyPhen-2, FAT – FATHMM. Percentage of agreements of predicted pathogenicity between softwares were represented in green while percentage of disagreements were represented in red.

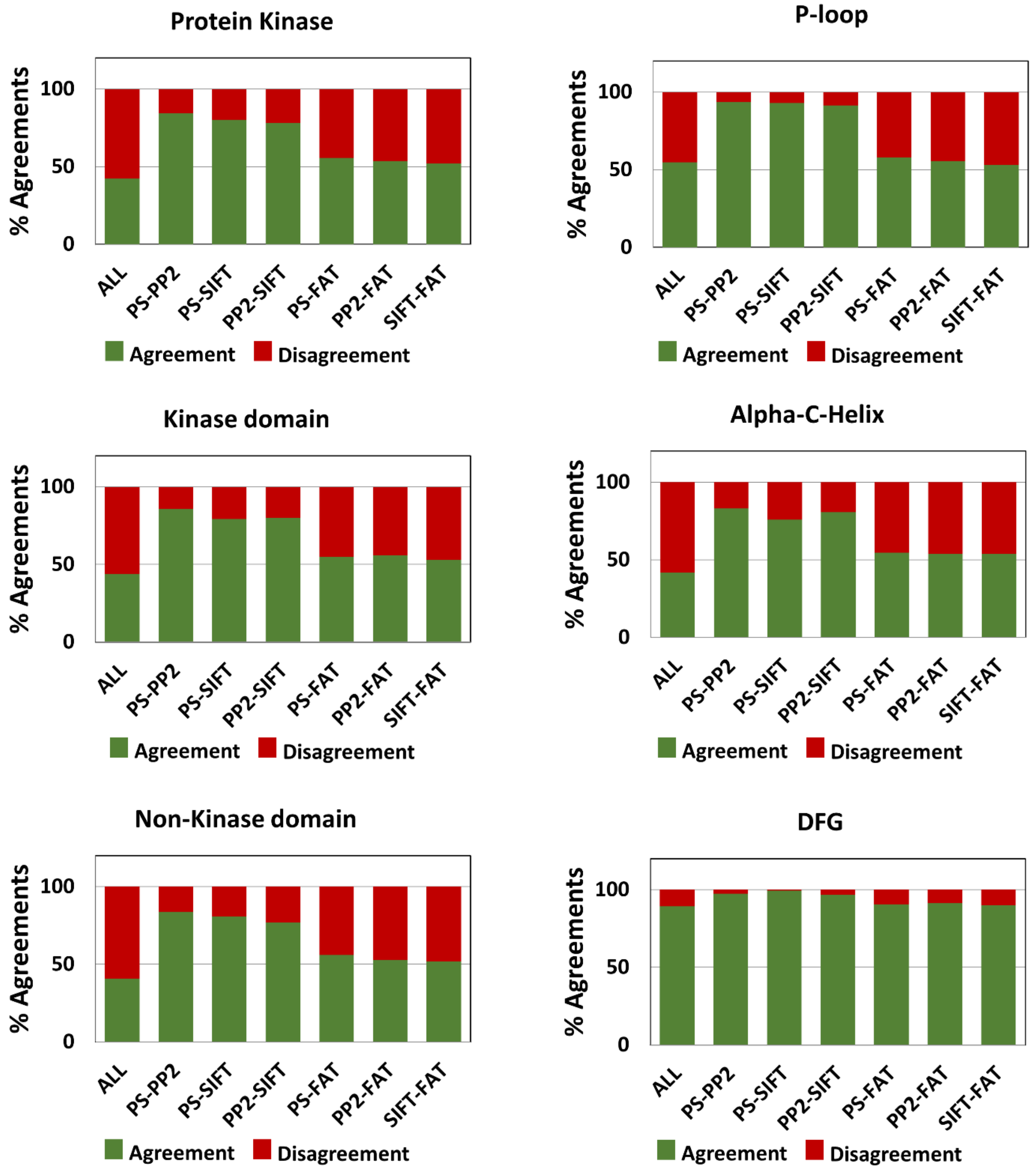

Supplementary figure 5: Variable degree of pathogenicity of mutations in individual kinases. (A) Consensus-pathogenicity profiles for individual kinases within different groups. The colours within bars represents the percentage of mutations predicted to be deleterious/cancerous in each kinase by primary structure-based tools. Blue: percentage of mutations predicted to be deleterious/cancerous by all the four softwares; orange: percentage of mutations predicted to be deleterious/cancerous by any three softwares; yellow: percentage of mutations predicted to be deleterious by any two softwares among PS, PP2 and SIFT or cancerous by FATHMM; white: percentage of mutations predicted to be deleterious by any one software among PS, PP2 and SIFT or neutral by all four softwares.

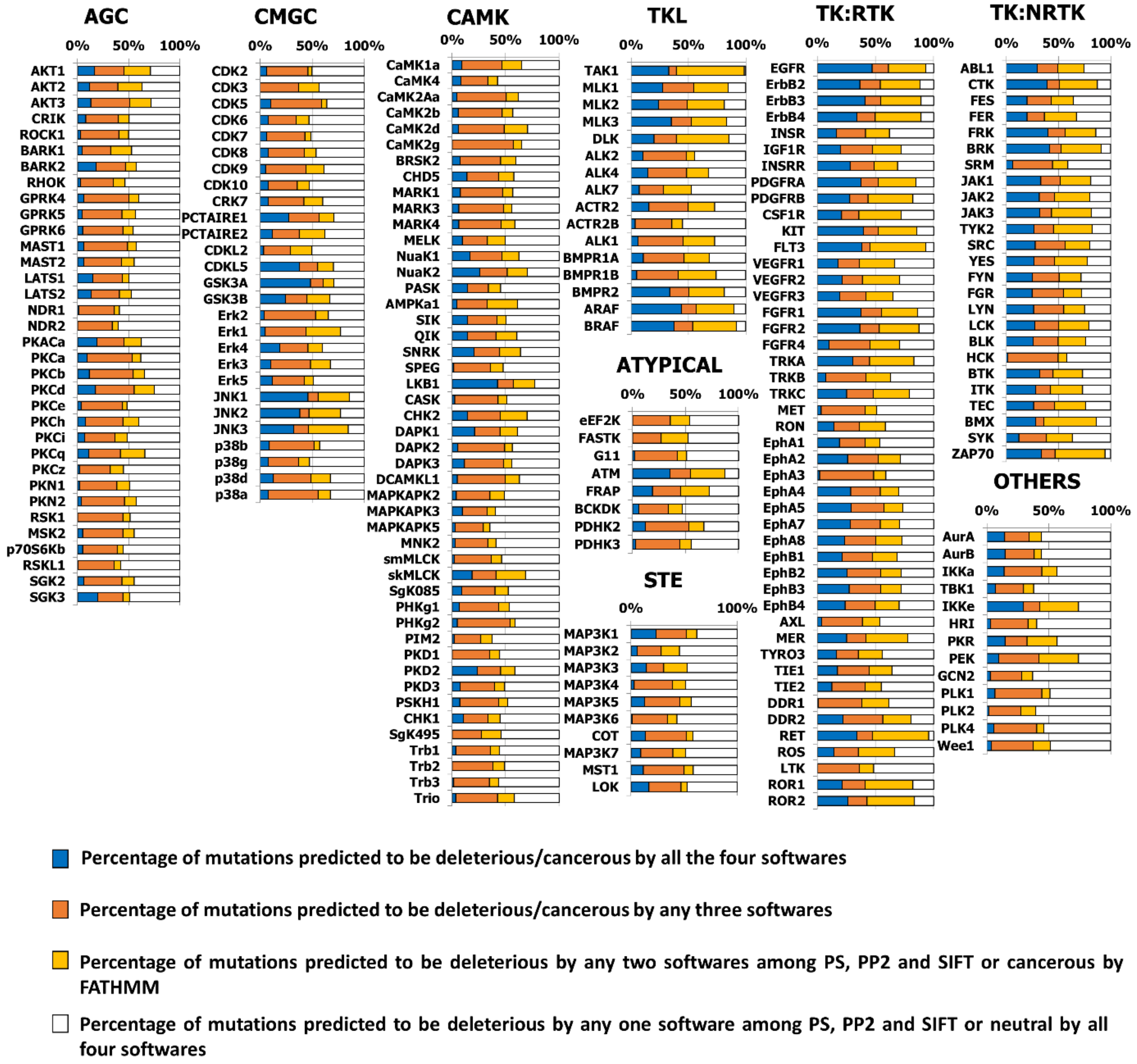

Supplementary figure 6: Data set of 141 kinase mutations (99 cancerous and 42 inert) with experimentally proven activity. (A) Predictions of primary and tertiary structure-based tools for experimentally proven cancerous mutations as evidenced by their transformation potential of cytokine-dependent Ba/F3 cells. The strength of prediction was shown in shades of black: light grey for weak to black for strong prediction. For primary structure-based prediction, consensus of all the tools were considered. For tertiary structure-based tools, destabilizing effect was considered. (B) List of kinase impaired mutants and missense SNPs used in the current study.

A.

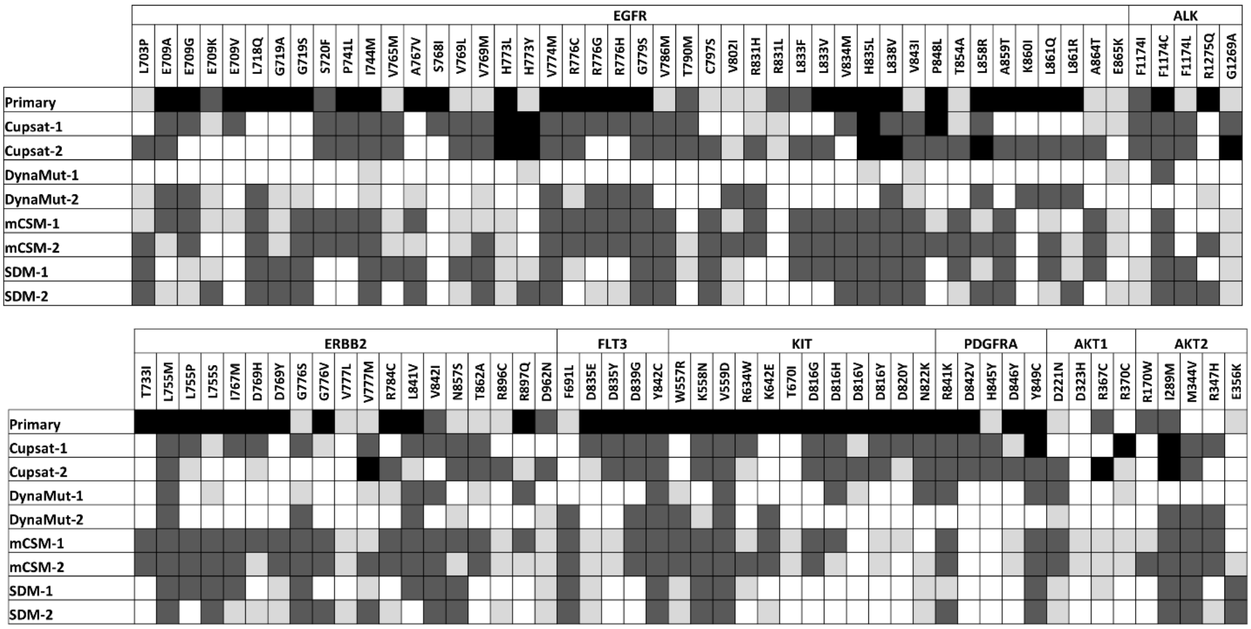

B.

ALK: V476A, Q500H, T680I, H1030L, H1030P, K1491R, K1491T, D1529E
ATR: D2494E
BRAF: K483M, D594A, D594V
EGFR: R521K, R521M, R521T, L688P, 787H, D837N, R841M, V851A, I853T, E866K
ERBB2: I655F, I655V, D1058A, P1170A, P1170S, P1170T
FLT3: D7G, T227K, T227M, D324N, D324Y, V557I
KIT: M541L, M541V
LRRK2: A1904F, K1906M, D2017A
PDGFRA: S478P, S478T
TRKA: K544N
